# Poly[(μ_4_-1,2,3-benzothia­diazole-7-carboxyl­ato)silver(I)]

**DOI:** 10.1107/S1600536810027029

**Published:** 2010-07-21

**Authors:** Jiong-Peng Zhao, Fu-Chen Liu

**Affiliations:** a School of Chemistry and Chemical Engineering, Tianjin University of Technology, Tianjin 300191, People’s Republic of China

## Abstract

In the crystal structure of the title compound, [Ag(C_7_H_3_N_2_O_2_S)]_*n*_, the Ag^I^ atom is coordinated by two N atoms and three O atoms of four organic ligands forming a distorted square pyramid. The carboxyl­ate group acts as a bidentate ligand on one Ag^I^ atom and as a bridging group for a symmetry-related Ag^I^ atom, forming a dimer. Futhermore, the two N atoms of two thia­diazole rings bridge a third symmetry-related Ag^I^ atom, forming a six-membered ring. These two frameworks, AgO_2_Ag and AgN_4_Ag, extend in three directions, forming a three-dimensionnal polymer. The whole polymer is organized around inversion centers.

## Related literature

For a metal-organic complex with inter­esting properties, see: Yaghi *et al.* (2003 ▶). For related structures, see: Chen & Mak (2005 ▶); Ng & Othman (1997 ▶); Brammer *et al.* (2002 ▶). 
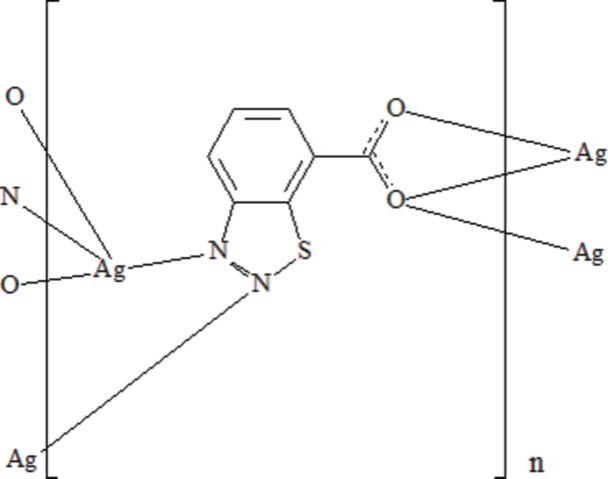


## Experimental

### 

#### Crystal data

[Ag(C_7_H_3_N_2_O_2_S)]
*M* *_r_* = 287.04
Monoclinic, 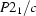

*a* = 5.8332 (12) Å
*b* = 14.786 (3) Å
*c* = 8.6377 (17) Å
β = 93.63 (3)°
*V* = 743.5 (3) Å^3^
*Z* = 4
Mo *K*α radiation
μ = 2.95 mm^−1^
*T* = 293 K
0.20 × 0.18 × 0.17 mm

#### Data collection

Rigaku SCXmini diffractometer
Absorption correction: multi-scan (*ABSCOR*; Higashi, 1995 ▶) *T*_min_ = 0.630, *T*_max_ = 1.000
6233 measured reflections
1291 independent reflections
1144 reflections with *I* > 2σ(*I*)
*R*_int_ = 0.044

#### Refinement

*R*[*F* ^2^ > 2σ(*F* ^2^)] = 0.041
*wR*(*F* ^2^) = 0.075
*S* = 1.16
1291 reflections
118 parameters
H-atom parameters constrained
Δρ_max_ = 1.26 e Å^−3^
Δρ_min_ = −0.63 e Å^−3^

### 

Data collection: *SCXmini Benchtop Crystallography System Software* (Rigaku, 2006 ▶); cell refinement: *PROCESS-AUTO* (Rigaku, 1998 ▶); data reduction: *PROCESS-AUTO*; program(s) used to solve structure: *SHELXS97* (Sheldrick, 2008 ▶); program(s) used to refine structure: *SHELXL97* (Sheldrick, 2008 ▶); molecular graphics: *ORTEPIII* (Burnett & Johnson, 1996 ▶) and *PLATON* (Spek, 2009 ▶); software used to prepare material for publication: *SHELXTL* (Sheldrick, 2008 ▶).

## Supplementary Material

Crystal structure: contains datablocks global, I. DOI: 10.1107/S1600536810027029/dn2580sup1.cif

Structure factors: contains datablocks I. DOI: 10.1107/S1600536810027029/dn2580Isup2.hkl

Additional supplementary materials:  crystallographic information; 3D view; checkCIF report

## Figures and Tables

**Fig. 1.**
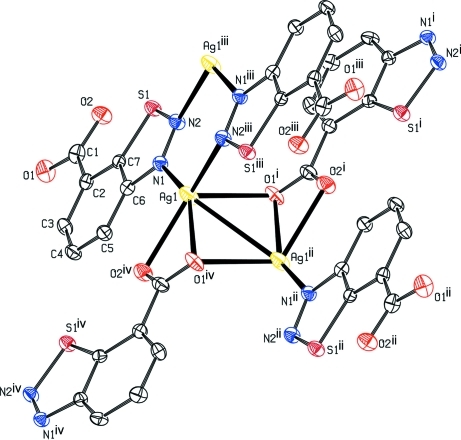
The coordinated mode of the metal ions. Ellipsoids are drawn at the 30% probability level. H atom have been omitted for clarity. [ Symmetry codes: i -x+1, y-1/2,-z+1/2; ii -x, -y+1,-z+1; iii -x+1,-y+1,-z+1; iv x-1,-y+3/2,z+1/2].

**Fig. 2.**
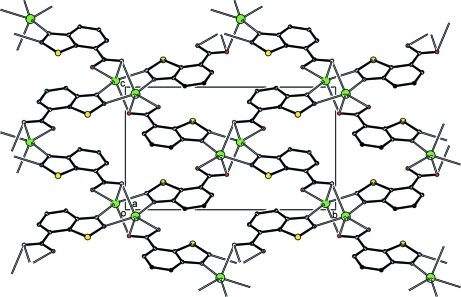
Packing view of the 3D structure viewed along the a axis. H atoms have been omitted for clarity.

## supplementary crystallographic information

### Comment

Metal organic complexes have drawn much attentions owing to their various structures and their interesting properties (Yaghi *et al.*, 2003). As a bridging ligand benzo[d][1,2,3]thiadiazole-7-carboxylate (L) with three types of heteroatoms has been less investigated. Here we reported the structure of the title complex.

In the title compound, Ag^I^ is coordinated by two N atoms and three oxygen atoms of four organic ligands forming a distorted square pyramid. The carboxylate group acts as a bidentate ligand on one silver atom and as a bridging group for a symmetry related silver forming a dimer. Futhermore the two nitrogen atoms of two thiadiazole rings bridge a third symmetry related Ag atom forming a six membered ring (Fig. 1). The Ag-O and Ag-N distances are in good agreement with the values observed in related Ag^I^ complexes (Chen *et al.*, 2005; Ng & Othman, 1997; Brammer *et al.*, 2002) . The thiadiazole groups bridge two Ag^I^ anions using two nitrogen atoms living the sulfur atoms uncoordinated. In the dimer formed by the carboxylate group, Ag···Ag distance is 3.1168 (12)Å.

The two frameworks AgO2Ag and AgN4Ag extend in the three direction to form a three dimensionnal polymer (Fig. 2) .The whole polymer is organised around inversion centers.

### Experimental

A mixture of Ag(I)nitrate (1.5mmol), benzo[d][1,2,3]thiadiazole-7-carboxylate acid (0.75 mmol), in 10 ml water solvent was sealed in a Teflon-lined stainless-steel Parr bomb that was heated at 413 K for 48 h. Red crystals of the title complex were collected after the bomb was allowed to cool to room temperature.Yield 20% based on metal salte.

### Refinement

Hydrogen atoms were included in calculated positions and treated as riding on their parent C atoms with C—H = 0.93Å and *U*_iso_(H) = 1.2*U*_eq_(C).

### Crystal data

**Table:**

[Ag(C_7_H_3_N_2_O_2_S)]	*F*(000) = 552
*M**_r_* = 287.04	*D*_x_ = 2.564 Mg m^−^^3^
Monoclinic, *P*2_1_/*c*	Mo *K*α radiation, λ = 0.71073 Å
Hall symbol: -P 2ybc	Cell parameters from 6859 reflections
*a* = 5.8332 (12) Å	θ = 3.5–27.7°
*b* = 14.786 (3) Å	µ = 2.95 mm^−^^1^
*c* = 8.6377 (17) Å	*T* = 293 K
β = 93.63 (3)°	Block, yellow
*V* = 743.5 (3) Å^3^	0.2 × 0.18 × 0.17 mm
*Z* = 4	

### Data collection

**Table:**

Rigaku SCXmini diffractometer	1291 independent reflections
Radiation source: fine-focus sealed tube	1144 reflections with *I* > 2σ(*I*)
graphite	*R*_int_ = 0.044
ω scans	θ_max_ = 25.0°, θ_min_ = 3.5°
Absorption correction: multi-scan (*ABSCOR*; Higashi, 1995)	*h* = −6→6
*T*_min_ = 0.630, *T*_max_ = 1	*k* = −17→17
6233 measured reflections	*l* = −10→10

### Refinement

**Table:**

Refinement on *F*^2^	Primary atom site location: structure-invariant direct methods
Least-squares matrix: full	Secondary atom site location: difference Fourier map
*R*[*F*^2^ > 2σ(*F*^2^)] = 0.041	Hydrogen site location: inferred from neighbouring sites
*wR*(*F*^2^) = 0.075	H-atom parameters constrained
*S* = 1.16	*w* = 1/[σ^2^(*F*_o_^2^) + (0.0199*P*)^2^ + 2.3752*P*] where *P* = (*F*_o_^2^ + 2*F*_c_^2^)/3
1291 reflections	(Δ/σ)_max_ = 0.001
118 parameters	Δρ_max_ = 1.26 e Å^−^^3^
0 restraints	Δρ_min_ = −0.63 e Å^−^^3^

### Special details

**Table:**

Geometry. All esds (except the esd in the dihedral angle between two l.s. planes) are estimated using the full covariance matrix. The cell esds are taken into account individually in the estimation of esds in distances, angles and torsion angles; correlations between esds in cell parameters are only used when they are defined by crystal symmetry. An approximate (isotropic) treatment of cell esds is used for estimating esds involving l.s. planes.
Refinement. Refinement of *F*^2^ against ALL reflections. The weighted *R*-factor *wR* and goodness of fit *S* are based on *F*^2^, conventional *R*-factors *R* are based on *F*, with *F* set to zero for negative *F*^2^. The threshold expression of *F*^2^ > σ(*F*^2^) is used only for calculating *R*-factors(gt) *etc*. and is not relevant to the choice of reflections for refinement. *R*-factors based on *F*^2^ are statistically about twice as large as those based on *F*, and *R*- factors based on ALL data will be even larger.

### Fractional atomic coordinates and isotropic or equivalent isotropic displacement parameters (Å^2^)

**Table:**

	*x*	*y*	*z*	*U*_iso_*/*U*_eq_	
Ag1	0.22834 (8)	0.54824 (3)	0.55500 (6)	0.04253 (19)	
S1	0.8176 (2)	0.67965 (9)	0.27803 (16)	0.0309 (3)	
O2	0.9762 (7)	0.8433 (3)	0.1625 (4)	0.0393 (10)	
N1	0.4925 (7)	0.6364 (3)	0.4372 (5)	0.0285 (10)	
O1	0.8634 (7)	0.9824 (3)	0.2134 (5)	0.0437 (11)	
N2	0.6579 (8)	0.6012 (3)	0.3649 (5)	0.0305 (11)	
C1	0.8499 (10)	0.8983 (4)	0.2247 (6)	0.0341 (13)	
C5	0.3213 (9)	0.7828 (4)	0.4920 (6)	0.0303 (13)	
H5A	0.2098	0.7576	0.5511	0.036*	
C2	0.6652 (9)	0.8593 (4)	0.3167 (6)	0.0268 (12)	
C7	0.6539 (8)	0.7651 (3)	0.3390 (6)	0.0237 (11)	
C6	0.4826 (9)	0.7294 (3)	0.4252 (6)	0.0257 (12)	
C3	0.5022 (9)	0.9111 (4)	0.3794 (6)	0.0315 (13)	
H3A	0.5038	0.9732	0.3631	0.038*	
C4	0.3315 (9)	0.8741 (4)	0.4679 (6)	0.0328 (13)	
H4A	0.2246	0.9119	0.5104	0.039*	

### Atomic displacement parameters (Å^2^)

**Table:**

	*U*^11^	*U*^22^	*U*^33^	*U*^12^	*U*^13^	*U*^23^
Ag1	0.0450 (3)	0.0259 (3)	0.0598 (3)	−0.0019 (2)	0.0278 (2)	−0.0036 (2)
S1	0.0320 (8)	0.0274 (7)	0.0347 (8)	0.0004 (6)	0.0123 (6)	−0.0004 (6)
O2	0.039 (2)	0.041 (2)	0.040 (2)	0.0007 (19)	0.0199 (19)	0.0042 (19)
N1	0.028 (2)	0.023 (2)	0.034 (3)	−0.0008 (19)	0.005 (2)	−0.001 (2)
O1	0.057 (3)	0.033 (2)	0.043 (3)	−0.014 (2)	0.016 (2)	0.0093 (19)
N2	0.032 (3)	0.022 (2)	0.038 (3)	−0.001 (2)	0.006 (2)	0.002 (2)
C1	0.037 (3)	0.036 (3)	0.030 (3)	−0.012 (3)	0.004 (3)	0.003 (3)
C5	0.025 (3)	0.028 (3)	0.039 (3)	−0.005 (2)	0.011 (2)	−0.004 (2)
C2	0.029 (3)	0.029 (3)	0.022 (3)	−0.004 (2)	0.000 (2)	−0.001 (2)
C7	0.022 (3)	0.026 (3)	0.023 (3)	−0.002 (2)	0.001 (2)	−0.002 (2)
C6	0.027 (3)	0.027 (3)	0.023 (3)	−0.002 (2)	0.001 (2)	0.001 (2)
C3	0.041 (3)	0.021 (3)	0.033 (3)	0.000 (2)	0.006 (3)	0.000 (2)
C4	0.031 (3)	0.028 (3)	0.041 (3)	0.002 (2)	0.010 (3)	−0.005 (3)

### Geometric parameters (Å, °)

**Table:**

Ag1—N1	2.304 (4)	C1—C2	1.494 (7)
Ag1—N2^i^	2.396 (4)	C5—C4	1.367 (7)
Ag1—O2^ii^	2.402 (4)	C5—C6	1.383 (7)
Ag1—O1^iii^	2.540 (4)	C5—H5A	0.9300
Ag1—Ag1^iv^	3.1168 (12)	C2—C3	1.360 (7)
S1—C7	1.688 (5)	C2—C7	1.408 (7)
S1—N2	1.693 (4)	C7—C6	1.388 (7)
O2—C1	1.242 (7)	C3—C4	1.404 (7)
N1—N2	1.292 (6)	C3—H3A	0.9300
N1—C6	1.379 (6)	C4—H4A	0.9300
O1—C1	1.251 (7)		
			
N1—Ag1—N2^i^	117.94 (15)	O2—C1—C2	116.4 (5)
N1—Ag1—O2^ii^	103.64 (15)	O1—C1—C2	118.4 (5)
N2^i^—Ag1—O2^ii^	132.00 (14)	C4—C5—C6	117.6 (5)
N1—Ag1—O1^iii^	85.52 (15)	C4—C5—H5A	121.2
N2^i^—Ag1—O1^iii^	87.08 (14)	C6—C5—H5A	121.2
O2^ii^—Ag1—O1^iii^	120.64 (14)	C3—C2—C7	117.5 (5)
N1—Ag1—Ag1^iv^	135.00 (11)	C3—C2—C1	122.7 (5)
N2^i^—Ag1—Ag1^iv^	83.21 (11)	C7—C2—C1	119.7 (5)
O2^ii^—Ag1—Ag1^iv^	83.74 (10)	C6—C7—C2	119.4 (5)
O1^iii^—Ag1—Ag1^iv^	54.53 (10)	C6—C7—S1	108.9 (4)
C7—S1—N2	92.1 (2)	C2—C7—S1	131.7 (4)
C1—O2—Ag1^v^	97.2 (3)	N1—C6—C5	124.4 (5)
N2—N1—C6	113.3 (4)	N1—C6—C7	113.1 (4)
N2—N1—Ag1	121.7 (3)	C5—C6—C7	122.6 (5)
C6—N1—Ag1	124.8 (3)	C2—C3—C4	122.4 (5)
C1—O1—Ag1^vi^	116.2 (4)	C2—C3—H3A	118.8
N1—N2—S1	112.7 (3)	C4—C3—H3A	118.8
N1—N2—Ag1^i^	115.7 (3)	C5—C4—C3	120.4 (5)
S1—N2—Ag1^i^	127.6 (2)	C5—C4—H4A	119.8
O2—C1—O1	125.1 (5)	C3—C4—H4A	119.8

Symmetry codes: (i) −*x*+1, −*y*+1, −*z*+1; (ii) *x*−1, −*y*+3/2, *z*+1/2; (iii) −*x*+1, *y*−1/2, −*z*+1/2; (iv) −*x*, −*y*+1, −*z*+1; (v) *x*+1, −*y*+3/2, *z*−1/2; (vi) −*x*+1, *y*+1/2, −*z*+1/2.
